# Neuromorphic Vibrotactile Stimulation of Fingertips for Encoding Object Stiffness in Telepresence Sensory Substitution and Augmentation Applications

**DOI:** 10.3390/s18010261

**Published:** 2018-01-17

**Authors:** Francesca Sorgini, Luca Massari, Jessica D’Abbraccio, Eduardo Palermo, Arianna Menciassi, Petar B. Petrovic, Alberto Mazzoni, Maria Chiara Carrozza, Fiona N. Newell, Calogero M. Oddo

**Affiliations:** 1 Sant’Anna School of Advanced Studies, The BioRobotics Institute, 56025 Pisa, Italy; luca.massari@santannapisa.it (L.M.); jessica.dabbraccio@santannapisa.it (J.D.); arianna.menciassi@santannapisa.it (A.M.); alberto.mazzoni@santannapisa.it (A.M.); chiara.carrozza@santannapisa.it (M.C.C.); 2 Department of Mechanical and Aerospace Engineering, “Sapienza” University of Rome, 00185 Roma, Italy; eduardo.palermo@uniroma1.it; 3 Production Engineering Department, Faculty of Mechanical Engineering, University of Belgrade, 11120 Belgrade, Serbia; pbpetrovic@mas.bg.ac.rs; 4 Academy of Engineering Sciences of Serbia (AISS), 11120 Belgrade, Serbia; 5 School of Psychology and Institute of Neuroscience, Trinity College, 2 Dublin, Ireland; fnewell@tcd.ie

**Keywords:** tactile perception, neuromorphic, contingency-mimetics, telepresence, vibrotactile, sensory augmentation, haptics

## Abstract

We present a tactile telepresence system for real-time transmission of information about object stiffness to the human fingertips. Experimental tests were performed across two laboratories (Italy and Ireland). In the Italian laboratory, a mechatronic sensing platform indented different rubber samples. Information about rubber stiffness was converted into on-off events using a neuronal spiking model and sent to a vibrotactile glove in the Irish laboratory. Participants discriminated the variation of the stiffness of stimuli according to a two-alternative forced choice protocol. Stiffness discrimination was based on the variation of the temporal pattern of spikes generated during the indentation of the rubber samples. The results suggest that vibrotactile stimulation can effectively simulate surface stiffness when using neuronal spiking models to trigger vibrations in the haptic interface. Specifically, fractional variations of stiffness down to 0.67 were significantly discriminated with the developed neuromorphic haptic interface. This is a performance comparable, though slightly worse, to the threshold obtained in a benchmark experiment evaluating the same set of stimuli naturally with the own hand. Our paper presents a bioinspired method for delivering sensory feedback about object properties to human skin based on contingency–mimetic neuronal models, and can be useful for the design of high performance haptic devices.

## 1. Introduction

The use of haptic technologies to deliver tactile information in real-time has grown in recent years, because of the effectiveness of the human tactile sense as a communication channel for different kinds of object information. We use the sense of touch to interact with the surrounding world, especially through the hands, which represent the most somatosensitive part of our body [1,2]. Individuals who have experienced the loss of this sense cannot properly perform actions, such as using tools or holding objects. For these persons, motor control tasks can also become extremely difficult or impossible to perform [3,4].

The possibility to deliver tactile information directly onto the users’ skin, and especially on the hands, via tactile feedback can enhance the performance of tasks executed in different scenarios. This could contribute, amongst others, to rehabilitation procedures, as well as to the development of tactile aids for visual and audition sensory disabled persons since the seminal works of Bach-y-Rita [5,6,7,8,9], as it has been discussed in perspective and review papers on the topic [10,11,12]. These individuals could improve their communication and navigation abilities through the use of wearable or hand-held haptic aids, particularly those which deliver information related to another sense via the tactile channel [12]. The delivery of tactile information is also fundamental in contexts where fine control of mechanical tools or robotic hands is required. More generally, the real-time representation of tactile information is highly important in virtual reality and telepresence contexts, where a user is allowed to perceive his/her presence in a virtual or remote real environment. Therefore, in order to reach a higher level of realism in these situations, visual and auditory stimuli shall be accompanied by haptic sensations with tactile feedback [13]. The stimulation of the tactile sense facilitates direct contact with virtually or remotely explored objects, providing the perception of characteristics such as weight, stiffness, thermal and geometric properties.

In the last decades, a variety of research has focused on the development of devices for the provision of tactile information in telepresence, mainly with sensory augmentation or substitution purposes [14,15,16,17]. However, there is still a significant gap between the wealth of research prototypes and the limited number of wearable haptic technologies that have reached a proper readiness level to turn into commercial devices.

Tactile information presented on the skin is collected by all the mechanoreceptors distributed in the stimulated areas and the dedicated receptors can then provide information about the characteristics of an object [18]. In general, the hand is the most selected area to be stimulated because receptors within the skin of human hands have the highest body density and are able to encode a relatively wide range of stimulation frequencies [19,20,21,22], with a concurrent role of the different receptor types [23,24]. Slowly adapting (SA) fibers (Merkel and Ruffini mechanoreceptors) respond dominantly to sustained stimuli with main frequencies up to 100 Hz [25], while rapidly adapting (RA) fibers (Meissner and Pacinian mechanoreceptors) are involved in the representation of vibrations and tickle, with activation frequencies reaching 200–300 Hz for Pacinian receptors [23]. The maximum spatial sensitivity is achieved on the index phalanx, where the location of the presented tactile stimulation is precisely encoded [26].

The delivery of tactile feedback in telepresence operations [27,28,29,30] is fundamental to augmenting users’ perception capabilities in scenarios such as industrial manufacturing. When robotic end-effectors are highly involved in both force and precision gripping tasks, there could be the possibility to improve high-precision manipulation activities with the development of human–robot control systems. Another important application is robotic surgery, where tactile feedback on a surgeon’s hand can transmit the characteristics of the inspected tissues [31,32,33]. In the last few years, much effort has been devoted to this field, for the development of systems for biomedical applications such as telemedicine and tele-robotics, and for the development of tactile sensors and actuators for non-structured environments [34]. In the last ten years, the advantages offered by medical robots for different procedures became clearer to the medical community [35]. A telling example is minimal invasive surgery (MIS), where the surgeon works out of the operating table and the manipulations are transmitted to the operation site by means of instruments [36]. In all these situations, a major drawback on performance is the absence of direct tactile feedback on the surgeon’s hands. In fact, tactile information is indicative of the condition of the operated tissues, but is also useful to ensure the correct manipulation of instruments [37]. An instrumented palpation would help to characterize tissues according to their elasticity and stiffness, to precisely localize tumors and lesions. For these purposes, some devices were developed in order to detect tumorous tissues [34], or to manipulate and characterize the properties of organs during robotics-assisted surgical interventions [38]. Some feedback mechanisms are being evaluated in laboratory environments in robotic surgery, like in the “Da Vinci” system (Intuitive Surgical Inc., Sunnyvale, CA, USA), where a force feedback system partially compensates for the absence of direct tactile feedback [34], or in which some tactile sensors were integrated in order to deliver tactile feedback on the surgeon’s hands via a pneumatic tactile display [34,39]. Force feedback devices such as the “Phantom” were also integrated in the “Zeus” surgical system [35]. Despite all these initial efforts, we are still far from a satisfactory delivery of tactile sensation in surgical environments.

In the present study, we tested the reliability of a tactile telepresence system in delivering feedback information about the stiffness, i.e., the ratio between a compression force and the resulting deformation of the tissue, of selected polymeric samples to the hand of an operator placed remotely with respect to the inspecting system. The study was designed with possible applications in the field of industrial and surgical robotics. Our approach is based on the conversion of information about the stiffness of rubber surfaces in vibrotactile stimuli by means of neuronal spiking models. This encoding strategy enables the real-time conversion of force information in neuronal spikes, which are delivered directly on the skin of the fingertips of the hand of a remote human subject. In our novel approach, the spikes, usually delivered via neuronal electric stimulation directly on nerves to restore tactile sensation in amputees [40] or to investigate how tactile information is encoded in the brain [41], are presented mechanically on the surface of the skin. The developed feedback strategy is based on a spiking neuron model and thus allows a discrete-events encoding of tactile information: scientifically; the proposed approach aims at merging together the simplification mechanisms of the discrete event-driven sensory feedback control (DESC) policy [42] with the possibility of delivering qualitatively-rich haptic information. Technically, the advantages of the proposed solution are the elegant and adaptable formulation via the differential equations that govern the Izhikevich artificial neuron, in place of the case-based programming that typically occurs with state machines in traditional systems. Another major technical advantage of the developed system is in its capability to reduce the bandwidth required to transfer and store information, as demonstrated in previous studies [43], with the potential advantages in enabling the streaming of data from large networks of tactile sensors in haptic telepresence applications. The main purpose of the present study is thus to investigate the mechanisms of tactile perception and the feasibility of the proposed feedback strategy, according to which vibrotactile information is delivered to the skin with an encoding strategy resembling the tactile receptor language, with the adaptation of models recently presented by our group [40,41,43]. The assumptions made in proposing this experimental paradigm are quite strong, because the temporal characteristics of the code delivered to the skin resemble the output—not the input—language of the receptors: this approach somehow resembles the contingency–mimetics strategy [44,45,46]. The scientific question addressed is then whether this language can be effectively understood even if delivered onto the skin surface rather than into the nerve (the approach typically pursued in prosthetics [40]).

The telepresence device is constituted of a mechatronic platform for the automatized indentation of rubber samples and of a haptic display (a vibrotactile glove) for the remote transmission of vibrotactile information [47]. Using a two-alternative-forced-choice (2-AFC) psychophysical protocol, we evaluated the reliability of this wearable haptic system in delivering stiffness information about remote objects in real-time.

## 2. Materials and Methods

### 2.1. Experimental Setup

**Figure 1:**
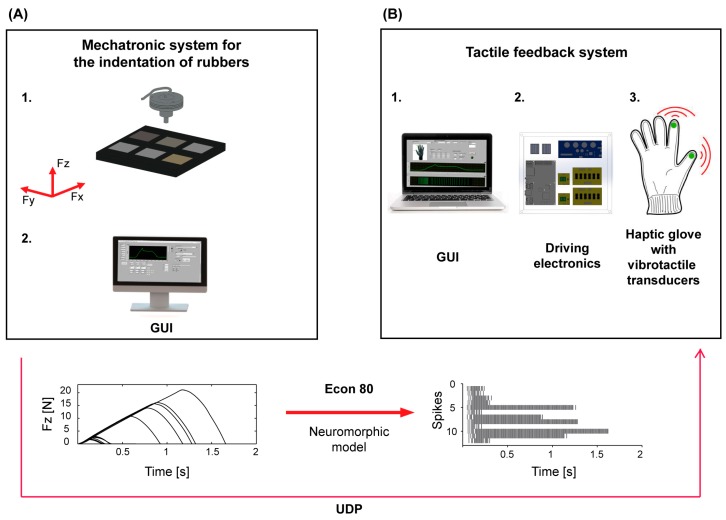
Experimental setup. (**A**) Subsystem I: 1. Sliders system with load cell and probe for the indentation of the rubber samples at controlled velocity; 2. Interface for the control of the platform operation and for sending data from the load cell to the second environment (remote laboratory); (**B**) Subsystem II: 1. Graphical User Interface (GUI) for the activation of the glove; 2. Driving electronics; 3. Haptic glove for the bi-digital stimulation of the hand. The two sub-setups were spatially separated, since the former was located in Italy and the latter in Ireland. The plots at the bottom of the figure depict how the normal force readings are converted into spikes by means of the implemented Izhikevich artificial neuron model.

The experimental setup was composed of two main subsystems, which allowed for the execution of the experiments in telepresence. The first subsystem was a mechatronic platform for the indentation of samples of different rubber surfaces. It consisted of a cartesian manipulator with three translational degrees of freedom, a load cell for the measurement of the normal force during the indentation of the samples (Nano 43, ATI Industrial Automation, Apex, NC, USA), a probe mounted on the load cell and a Graphical User Interface (GUI) made in LabView (National Instruments Corp., Austin, TX, USA) in order to control the movement of the sliders and acquire data from the load cell (Figure 1A). This first subsystem was located in a laboratory of The BioRobotics Institute of Sant’Anna School of Advanced Studies—Pisa, Italy.

The second sub-setup was located in a remote environment with respect to the indentation platform, i.e., a laboratory within the Trinity College Institute of Neuroscience in Dublin, Ireland. The apparatus comprised a textile glove equipped with two vibrotactile piezoelectric elements which were placed on the index and thumb fingertips respectively, a control electronics and a GUI made in LabView, which allowed the transmission of data from the mechatronic platform to the haptic glove (Figure 1B). The communication between the two blocks of the experimental setup was performed via a User Datagram Protocol (UDP) channel.

The mechatronic platform for the automatic indentation of the rubber samples (Figure 1A) allowed for the investigation of the participants’ discrimination thresholds in the experimental condition of passive touch, with the purpose of characterizing relevant psychophysical parameters. The platform allowed for the measurement of the normal force (*Fz*) generated during the rubber compression stage. In particular, it executed indentation protocols with controlled testing velocities and positions, and selectable force targets. The vertical element of the slider mechanism was a precision positioner, and could ensure the vertical application of loads up to 10 kg along the *z-*axis. The couple of sliders along the *x*- and *y*-axes had the same performance as the vertical positioner. The probe was fixed to the load cell: it was a cylindrical tip made of aluminum with a diameter of 6 mm.

During the indentation of the rubber samples, the vertical slider was driven with a constant velocity of 0.5 mm/s, which was always the same for each investigated specimen. The choice of a constant indentation velocity allowed us to characterize the stiffness of the samples without taking into account variations due to the viscoelastic behavior of the materials. In each experimental trial, the rubber pair, the force and the duration used to indent each rubber sample were randomized, in order to guarantee a very challenging task for the participants during the experimental protocol (see Section 2.3.1).

The tactile feedback system allowed for the vibrotactile stimulation on the remote user’s hand, giving information regarding the stiffness of the indented rubber samples (Figure 1B). It was composed of an electronic board (sbRIO 9636, National Instruments Corp., National Instruments, Austin, TX, USA) for the communication between the received force signal and the piezoelectric elements in the glove; a piezoelectric evaluation module (DRV2667 Evaluation module, Texas Instruments, Dallas, TX, USA) for the activation of the piezoelectric transducers; a vibrotactile interface for bi-digital stimulation, made of a textile glove embedding two piezoelectric disks (7BB-12-9, MuRata, Kyoto Prefecture, Japan) encapsulated in a polymeric matrix with a customized process [47,48].

The mechatronic platform moved the rubber samples in order to indent those selected for the current experimental session. The six rubber samples were placed on a Delrin support (170 × 130 × 17 mm) provided with six housings (30 × 30 × 3 mm^3^). The position of the center of each sample was represented by a couple of coordinates (*x-y*). These coordinates were randomized with a GUI made in LabView, which allowed for the movement of the horizontal sliders along the *x-y* axes. Once the target *x-y* position was reached, the translation mechanism allowed indenting the rubber via the probe along the *z*-axis. The rubber compression lasted until the measured *Fz* on the load cell reached a random threshold level, that was varied in the 4 to 8 N range during the experimental protocol. Once the threshold was reached, the loading phase was interrupted, and the support returned to the reference position (*x* = 0; *y* = 0; *z* = 0).

### 2.2. Spike-Based Encoding of Cutaneous Feedback Information via the Izhikevich Model

The activation of the piezoelectric transducers on subsystem II was triggered by a spiking neuron model which converted the normal force measured by the load cell on subsystem I (Figure 1) into spike trains. The implemented neuromorphic feedback strategy was based on a regular Izhikevich spiking model, where v and u represent the neuron membrane potential and recovery variable [49] (see (1)–(4) of the model). The Izhikevich spiking model was implemented via a GUI made in LabView (National Instruments). The parameters of the model were selected with a pilot evaluation to define a set of coefficients able to convert the measurements of the load cell into a train of spikes reflecting the magnitude and rate of change of the interaction force *F_z_* arising between the probe and the rubber samples $\left(F_{th}=0.08\;N;\;A=0.04\;{\mathrm{mS}}^{-1}{\mathrm{mV}}^{-1};\;B=5\;{\mathrm{ms}}^{-1};\;C=140\;{\mathrm{mVms}}^{-1};\;a=0.02\;{\mathrm{ms}}^{-1};\;b=0.2\;{\mathrm{ms}}^{-1};\;c=65\;\mathrm{mV};\;d=8\;\mathrm{mV};\;V_{th}=30\;\mathrm{mV};k=10\;{\mathrm{mVms}}^{-1}N^{-1}\right)$.

(1) $$\mathrm{If}\;\left(F_{z}-F_{th}\right)>0,\;\mathrm{then}\;F_{in}=\left(F_{z}-F_{th}\right),\;\mathrm{else}\;F_{in}=0$$

(2) $$\frac{dv}{dt}=Av^{2}+Bv+C-u+kF_{in}$$

(3) $$\frac{du}{dt}=a\left(bv-u\right)$$

and the after-spike resetting conditions:

(4) $$\mathrm{if}\;v\;\geq V_{th},\;\mathrm{then}\;\{\begin{array}{c} v\;\leftarrow c\\ u\;\leftarrow u+d \end{array}$$

**Figure 2:**
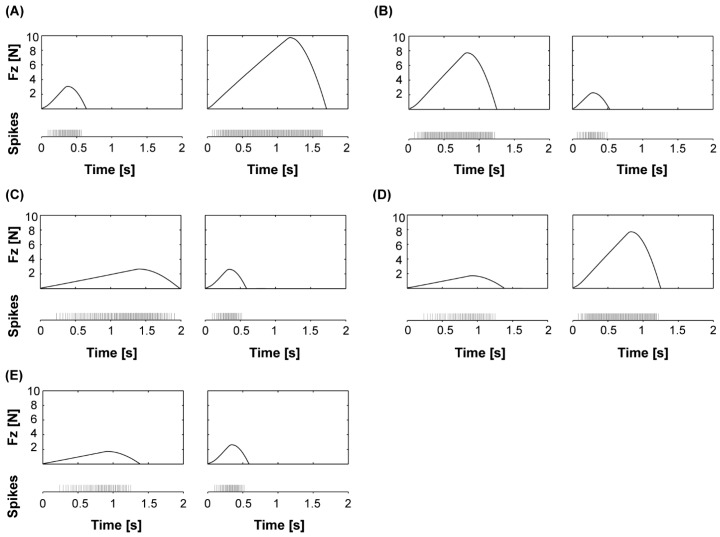
Example combinations of stimulus pairs presented in the experimental protocol. In each experimental trial, the rubber pair, the force and the duration used to indent each rubber sample were randomized, in order to guarantee a very challenging evaluation condition. The panels of this figure show some selected stimulation conditions. (**A**) Material 1 slightly stiffer than material 2: softer rubber sample indented with higher force (resulting in longer stimulation duration for the softer rubber sample); (**B**) Material 1 slightly stiffer than material 2: stiffer rubber sample indented with higher force (resulting in longer stimulation duration for the stiffer rubber sample); (**C**) Material 2 stiffer than material 1: both rubber samples indented with the same force (resulting in longer stimulation duration for the softer rubber sample); (**D**) Material 2 stiffer than material 1: indentation duration kept constant (resulting in stiffer rubber sample being indented with a higher force); (**E**) Material 2 stiffer than material 1: softer rubber sample indented with longer duration (resulting in lower level of indentation force for the softer rubber sample).

Figure 2 shows some examples of how rubber samples were combined during the psychophysical testing described in the following section (see Table 1 for selected combinations).

**Figure 3:**
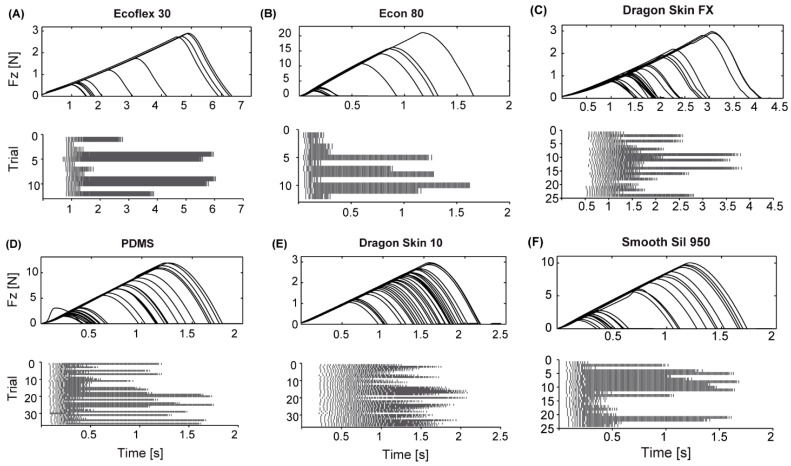
Conversion of the acquired normal forces in spike trains, per each selected rubber. The spikes were then delivered to the wearer of the haptic glove by means of encapsulated piezoelectric transducers. (**A**) Different indentations of the same rubber (Ecoflex 30) with different force threshold levels (upper panel), and corresponding spike trains per each rubber indentation (lower panel); (**B**) same of panel (**A**), for rubber Econ 80; (**C**) same of panel (**A**), for rubber Dragon Skin FX; (**D**) same of panel (**A**), for rubber Polydimethylsiloxane (PDMS); (**E**) same of panel (**A**), for rubber Dragon Skin 10; (**F**) same of panel (**A**), for rubber Smooth Sil 950. Spike activity was higher in the middle of the indentation, when the force level reached the threshold. The stimulation patterns shown in this figure were paired and presented to the participant according to the combination possibilities discussed in Figure 3. Note how the spike activity changed passing from a softer rubber (i.e., Ecoflex 30, in panel (**A**)) to a harder rubber (i.e., Econ 80, in panel (**B**)). Please note that in panel (**D**) (upper panel) there is a single non-coherent slope, probably an outlier of the measured *Fz* during the automatized indentation protocol.

The obtained spikes were then delivered to the glove by means of a piezoelectric driver (DRV2667, Texas Instruments), working in analog mode, so that the on–off activity of the transducers was regulated by the neuromorphic model, proportional to the magnitude of the normal force measured by subsystem I. The actuation parameters for the piezoelectric driver in analog mode were a 40.7 dB gain, a peak-to-peak voltage amplitude of 200 V and a Boost voltage of 105 V. Figure 3 shows example spike train patterns obtained for the rubber samples and indentation forces experimented during the psychophysical protocol.

### 2.3. Psychophysical Experiments

We evaluated the efficacy of the telepresence system in delivering reliable information regarding rubber stiffness using a two-alternative forced-choice (2-AFC) psychophysical protocol. Specifically, a control test was performed investigating an active touch condition naturally with the own hand, while the actual experimental protocol was focused on the investigation of the communication of stiffness information with neuromorphic tactile telepresence. The two protocols are detailed in the following.

An active touch experiment was performed in order to evaluate the benchmark performance of a cohort of participants who actively judged, via their own hand, the stiffness of the same rubber samples used in the telepresence experiment. This experimental protocol regarding active touch involved 10 participants, (five females and five males aged between 23 and 32, recruited from university students and staff of the BioRobotics Institute of the Sant’Anna School of Advanced Studies—Pisa). Participants self-reported having no pathologies regarding the tactile sense, nor was their tactile sensitivity compromised by previous activities. The tactile task involved the index fingertip of the dominant hand (one left-handed and nine right-handed). During the active touch experiment, participants were invited to take a seat and to place their dominant arm on a table. To avoid seeing the stimuli during the experiment, participants were also invited to insert their arm in a box where a cushion was placed for positioning the arm in a comfortable manner, with their palm facing down. During the task, the experimenter inserted in the box the holder containing the selected rubber stimuli, and guided the index fingertip of the participant’s dominant hand onto the two selected rubber samples, sequentially.

The tactile telepresence experiment involved ten participants (seven females and three males aged between 20 and 31, recruited from university students and staff of the Institute of Neuroscience Trinity College—Dublin). Participants self-reported having no pathologies regarding the tactile sense, nor was their tactile sensitivity compromised by previous activities. The tactile stimulation was performed on the participants reported dominant hand (one left-handed and nine right-handed).

The experiment was conducted in accordance with the Declaration of Helsinki, and the protocol was approved by the Ethics Committee for non-clinical experimentation of Sant’Anna School of Advanced Studies Pisa and by the Research Ethics Committee of the School of Psychology, Trinity College Dublin. All participants gave written informed consent.

During the telepresence experiment, each participant was invited to take a seat in a remote laboratory (Ireland) with respect to the one where the indentation platform was placed (Italy), and was invited to wear the vibrotactile glove on the dominant hand. Each participant was asked to wear a headset which provided white noise in order to mask the sound from the activation of the piezoelectric transducers.

#### 2.3.1. Benchmarking Protocol: Evaluation of the Stiffness of the Rubber Samples under Active Touch

**Figure 4:**
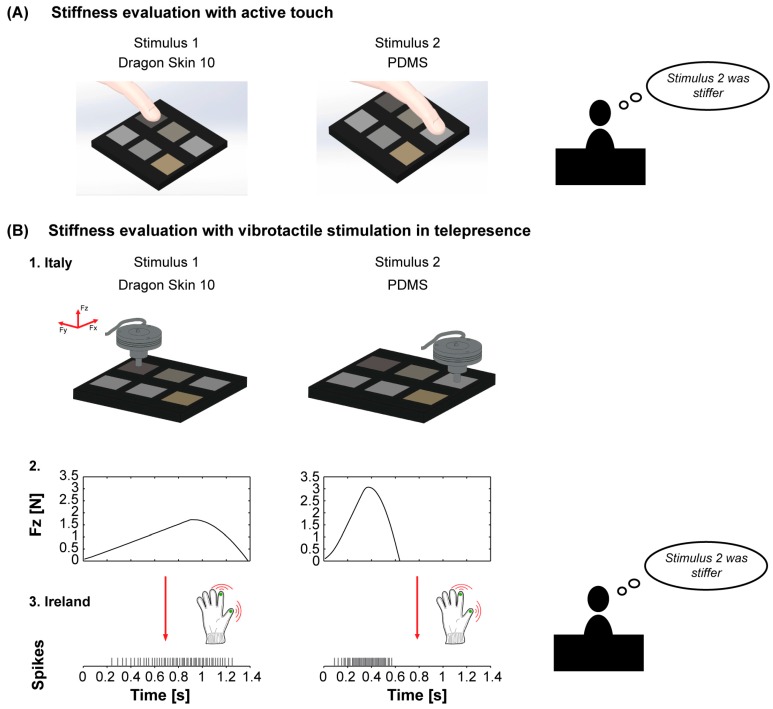
Experimental protocol for psychophysical experiments. (**A**) Stiffness evaluation with active touch. The participant index was guided towards the selected stimuli of the given couple. After the tactile evaluation, the participant was asked to judge which rubber of the pair was stiffer. (**B**) Stiffness evaluation with vibrotactile stimulation in telepresence. 1. The mechatronic platform, placed in a laboratory in Italy, indented the selected rubber of the given couple; 2. The measured normal force was converted in spikes using the Izhikevich artificial neuron spiking model; 3. The resulting spikes were delivered to the vibrotactile glove, placed in a remote laboratory in Ireland, for the haptic bi-digital stimulation of the participants’ hand. After the transmission of the spikes representing the second stimulus, the participant was asked to judge which rubber of the pair was stiffer.

The active touch experimental protocol was carried out for benchmarking purposes to evaluate the physiological acuity in discriminating stiffness variations. It consisted of a 2-AFC tactile discrimination task (Figure 4A). In each trial, the participant was provided with a pair of samples of rubber stimuli, characterized by different stiffness. The participant was asked to touch the center of each polymeric sample with the index fingertip of the dominant hand. In order to perform the task, the experimenter guided the index finger of the participant onto the first stimulus of the selected pair of stimuli, then rotated the lodging of the stimuli, and guided the finger of the participant onto the second stimulus. The participants were allowed to touch the stimuli as long as they needed and with the force they preferred in order to perceive the rubber stiffness. Participants were asked to provide a verbal evaluation of which stimulus of the presented pair was perceived as stiffer. For the active touch experiment, seven rubber samples were selected and combined in order to obtain six pairs in which normalized delta stiffness could be equally distributed along the psychophysical axis (see Table 1 for the list and properties of the selected stimuli). In the following analyses, the stiffness variation was normalized according to the mean of the calculated stiffness per each pair (as defined in Table 1), resulting in normalized stiffness variations comprised between 0.37 and 1.88. The experimental protocol consisted in the presentation of the six pairs of stimuli, in direct and reverse order, for a total of 12 trials (see Table 1). In this way, both the increasing and decreasing stiffness conditions were considered. The whole experiment consisted in the presentation of 72 trials in blocks of 12, with a 5 min break between the third and fourth block. The presentation of 12 trials took about 4 min, for a total duration of the experiment of about 20 min. Before each experimental session, every participant was presented with a training session, in order to familiarize them with the stimuli and the protocol. Each training session was about 5 min long and it consisted of touching all the samples in an increasing order of stiffness. All the participants understood the task with only one training session. A participant’s performance during these training sessions was not included in the main statistical analyses.

#### 2.3.2. Experimental Protocol: Evaluation of the Stiffness of the Rubber Samples via Spike-Based Vibrotactile Stimulation in Telepresence

The actual experimental protocol for evaluating the proposed spike-based cutaneous tactile feedback strategy consisted of a 2-AFC tactile discrimination task (Figure 4B). The participant was provided with pairs of vibrotactile signals in sequence, in which the timing of the vibrotactile spikes delivered by the glove was linked via the Izhikevich artificial neuron model to the amplitude of the normal force exerted on the load cell during the indentation of the samples, as described in Section 2.2. The participant was asked to give a verbal description of the perceived vibrotactile signals, identifying the stiffer rubber of the given pair of stimuli. In the telepresence task the participant received the stimuli fully under the control of the remote mechatronic platform, without the possibility to feel the pair of stimuli again.

Before an experimental session, each participant underwent a training session, in order to familiarize them with the stimulation apparatus and procedure. Each training session consisted of the presentation of six randomized trials, also provided in reverse order, for a total of 12 trials (Table 1). The trial session was about 15 min long. The performance of participants during these sessions was not included in the statistical analyses.

The whole experiment consisted in the presentation of 72 trials in blocks of 12, with a 5 min break between the third and fourth block in order to ensure concentration and avoid distress. In comparison with the active touch benchmarking protocol, the speed constraints of the mechatronic platform in the indentation of the stimuli, as well as in the transitions between the stimuli in a pair, enlarged the time required for the presentation of 12 trials, which took about 15 min. The duration of the total experiment was about 1 h and 30 min.

### 2.4. Data Analysis

Data analysis was performed using the Statistics Toolbox in Matlab (R2016b, MathWorks, Natick, MA, USA). For each stiffness variation, the success rate was evaluated across the population of participants, together with the 95% confidence interval (*binofit* test) of the rates of identification of stimuli with increasing stiffness (normalized Δstiffness > 0). A logistic fit of the resulting psychometric curves was computed for the presented stiffness variations using the Matlab *nlinfit* function, using a custom fitting cumulative distribution function. The significance of participants’ responses for each normalized stiffness variation (normalized Δstiffness) was computed using the Matlab *binofit* test.

## 3. Results

### Psychophysical Tests

**Figure 5:**
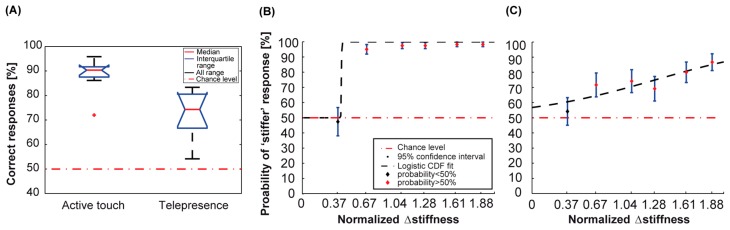
Psychophysics results of telepresence experiments. (**A**) Boxplot of correct responses for the two experimental conditions. Boxes represent interquartile range and black dashed lines show the complete range across participants. The red dashed line represents chance level; (**B**) Psychometric curve for the benchmarking psychophysics experiments with active touch. Each dot represents the fraction of times each stimulus was classified as having increasing stiffness (median across participants). If the identification rate is significantly different (probability of success > 50%, *binofit* test) from chance (50%) the dot is red, otherwise it is black. Error bars indicate the 95% confidence interval across participants and the red horizontal line represents chance. Black dashed line represents the logistic cumulative distribution function (CDF) fit; (**C**) Same as (**B**) for the psychophysics experiments in telepresence.

In the benchmark active touch experiment, we evaluated the capability of the human fingertip to perceive stiffness variations across pairs of rubber stimuli. To do so, experiments were performed according to a 2-AFC experimental protocol, with a sample of 10 volunteers. In the active touch condition, the discrimination performance was above 95% for all the stiffness variations, except for the stimulus closest to the origin, for which the stiffness variation was small (e.g., the pair PDMS/Smooth Sil 950 as showed in Table 1). The discrimination of each rubber stiffness by the active touch volunteers showed an average 90 ± 2% of correct responses over the whole range of stiffness variations (Figure 5A).

In the telepresence experiment, we assessed how the spike-based stimulation delivered through the glove enabled a sensory feedback for the stiffness of the rubber samples. A 2-AFC experimental protocol was used with a sample of 10 volunteers. The stimulus stiffness was encoded by the temporal pattern of spikes during the vibrotactile stimulation delivered to the fingers of the telepresence participants. For the telepresence condition, the discrimination performance was above 85% for large stiffness variations. The performance in discriminating the stiffness of the experimented rubber samples by the telepresence participants showed an overall average of 74 ± 7% (Figure 5A).

In both active- and telepresence-touch conditions, all stiffness variations were identified significantly better than by chance (probability of success > 50% in a binofit test of 10 participants), except for the first stimulus of the explored range of stiffness variation, for which the stiffness variation was small (Figure 5B,C and Table 1). In both the experimental conditions, the participant’s response was given by chance only when the pair with normalized Δstiffness = 0.37 (stimuli of comparable stiffness) was presented.

The experimental data were well-fitted by the symmetric logistic cumulative distribution function (CDF) (5), suggested by Ulrich and Miller [50], over the whole range of stiffness variations inspected in the test configuration:

(5) $$G\left(x\right)=0.5+0.5{\left[1+e^{\frac{-x-a}{b}}\right]}^{-1}$$

The fitting function denotes the probability of a correct response at difference *x*, where a represents the perceptual threshold and b>0 a scale parameter that affects the steepness of the curve. The experimental data were well fitted by the logistic CDF fit over the whole range of stiffness variations (see the black dashed line in Figure 5B,C). According to this model, the perceptual threshold above which participants could discriminate the difference in normalized stiffness of the rubber stimuli was a = 0.64 for the active touch condition, and a = 1.28 for the telepresence condition. The curve relative to the telepresence experiment displayed lower performance in comparison to the one relative to the active touch condition, for normalized Δstiffness > 0.37. The overall difference in performance between the telepresence and active touch benchmark experimental conditions (Figure 5) corresponded indeed to an effect size of 2.1 (corrected Hedge’s g) [51]. This suggests a better performance when the stiffness discrimination task was performed with the same set of stimuli in the active touch benchmark, naturally with the human hand. However, the two logistic fits showed more similar behavior when normalized Δstiffness was far from the values close to the origin of the psychometric curve (|normalized Δstiffness|>0.67, see Table 1), with the effect size (corrected Hedge’s g) of the difference between the two groups being 1.1 for |normalized Δstiffness|=1.88. Furthermore, considering that in the telepresence condition the rubber stiffness was converted to vibrotactile stimulation while both the indentation force and duration were randomized and not a-priori known by the participants, we can state that the telepresence task was more challenging than the benchmark active touch one.

## 4. Discussion and Conclusions

The goal of the present work was to evaluate the capability of a haptic system, a vibrotactile glove, in delivering reliable information about rubber stiffness in a telepresence configuration using a neuronal spiking model to trigger the delivery of stimuli. We investigated this topic using customized piezoelectric transducers embedded in a textile glove for the simultaneous stimulation of index and thumb fingertips. In order to do so, we compared the results from the vibrotactile stimulation of the hand with the benchmark experimental data relative to the active exploration of the rubber stimuli performed with the index fingertip.

### 4.1. Main Findings of the Study

We analyzed the reliability of a telepresence system in delivering, directly on the skin of a remote hand, haptic information about the stiffness of selected polymeric samples. The stimuli were indented by an automated system and their stiffness encoded according to a neuronal spiking model heuristically adapted from our previous implementations that emulate the firing activity of human mechanoreceptors [40,41,43]. This approach has some analogies with what has been termed contingency-mimetics, that applies biomimicry at the level of the sensory organ instead of the nerve [44,45,46]. We showed that the application of spike-based vibrotactile stimuli on the hand enabled the remote discrimination of most of the selected pairs of stimuli characterized by different stiffnesses. The stiffness discrimination performance was achieved by means of the proposed spike-based encoding of tactile information and was compared with that obtained via direct active touch exploration. During active touch exploration, participants were allowed to directly touch the rubber stimuli with no restrictions to the force and the duration of the contact, that were under human voluntary control. In the very challenging telepresence conditions created according to the indentation protocol, the indentation force and the duration of the contact were completely randomized and not known to the participants.

We acknowledge the several differences between the two experimental conditions: the time taken by the task (20 min for the active task vs. 90 for the telepresence task), the active vs. passive approach, the auditory masking (absent in the active task), and the control of the force and indentation velocity for the telepresence vs. no control in the active task. Despite this, with our work we aimed at demonstrating the proof of principle of a telepresence task, where tactile feedback can be representative of object characteristics (i.e., stiffness). Therefore, we preferred to design our experiment so that the proposed method was assessed in more challenging conditions than the natural benchmark. This study will be complemented with future research where participants will actively control a robotic arm in immersive telepresence, mimicking an active industrial task while receiving tactile information about the robot contact forces and displacement, whereas with typical virtual-reality-based human–machine interfaces the human operator can hardly recognize states when the robot has established contact with the objects in its environment.

**Figure 6:**
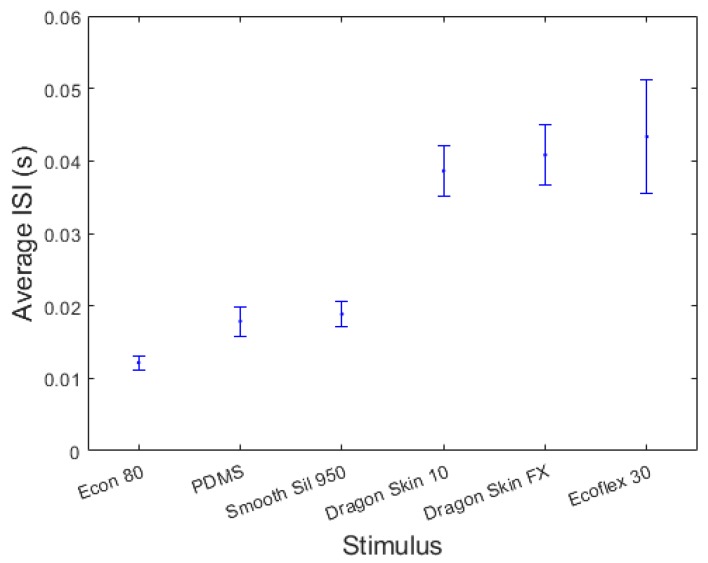
Experimented stimuli sorted from the stiffer to the more compliant, and related inter-spike interval. Error bars show the standard deviation of the inter-spike interval (ISI).

**Figure 7:**
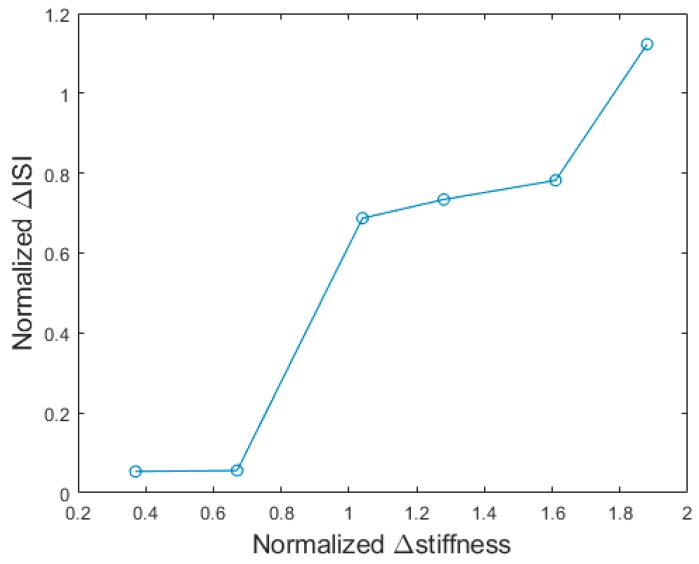
Normalized variation of the average inter-spike interval generated by the developed neuromorphic haptic interface versus the normalized variation of stiffness per each pair of experimented rubber samples (see Table 1 for the associations).

In order to deepen the explanation of the physical determinants of the responses given by the participants, we calculated the average inter-spike interval (ISI) for each rubber stimulus in the initial phase of the stimulation (calculated in a window of 120 ms after the first spike, that guarantee at least three spikes to be considered per each stimulus; see raster plots in Figure 3). Though with non-perfect separation between different rubber samples, the analysis demonstrated an inverse correlation between the ISI and the stiffness (Table 2; Figure 6), meaning that stiffer stimuli result in lower ISI (or more compliant ones result in higher ISI). This is because the force varies in a steeper manner in a stiffer stimulus while being indented at a constant velocity. A complementary analysis to investigate the psychophysical results was the estimation of the normalized ISI variation (defined as the normalized stiffness variation; see Table 1) as a function of the normalized stiffness variation within each pair of stimuli experimented via the developed neuromorphic haptic interface (Figure 7): a monotonic trend can be appreciated, however very low normalized Δ ISI were achieved at low normalized Δ stiffness. This explains the non-significant discrimination of the developed neuromorphic haptic interface while being used to remotely touch rubber samples with similar stiffness (see Figure 5c). Accordingly, future improvements of the system should aim at tuning the coefficients of the differential Equations (1)–(4) of the developed neuromorphic haptic interface, to amplify the ISI differences particularly with rubber samples of similar stiffness.

### 4.2. Potential Applications of Multi-Finger Vibrotactile Stimulation

Tactile information provided with haptic devices can partially compensate for a missing sense. Sensory substitution can be fundamental in cases of persons with blindness or visual impairment, deafness or hearing impairment and combined sensory impairment (deaf–blind). In these situations, the information coming from one sensory channel can be conveyed to the tactile sense in a perceptible manner [9,22,42,52,53] to allow, as an example, obstacle avoidance in assisted walking or remote communication with vibrotactile patterns. However, we are also aware of the intrinsic limitations in the possibilities to integrate perceptually (not only cognitively) a substitute sense in the own natural sensory scheme, as discussed in the work of Deroy and Auvray [10]. Vibrotactile stimulation can also be widely used in the field of non-invasive feedback in amputees and sensory augmentation for healthy subjects in applications such as virtual reality, gaming, rehabilitation, navigation, rescue and remote control of robots [27,28,29,30,31]. Examples of tactile devices for sensory augmentation providing whisker-type distance information were developed in the shape of head mounted systems [54,55,56] or belts [57,58] to improve navigation, as well as handheld systems for the detection of distances [58].

Tactile information was proven useful also in industrial environments for human–robot co-working activities. The development of alerting haptic devices, in environments where the interaction with automated machinery can be dangerous for operators, may also improve safety and the adoption of collaborative robotics in common applications.

Our tactile device can be classified within state of the art technologies where haptic feedback is used to provide sensory augmentation, with a particular focus on the remote perception of stiffness and potential applications in several fields, including robotic surgery. Our preliminary experimental protocol followed a simplified characterization approach for the rubber samples. Since the stiffness of the specimens was investigated via a constant indentation velocity of the probe, we did not consider variations in dynamic deformation behavior due to the viscoelastic characteristics of the rubber samples, which is an important aspect for the tactile characterization of a biological tissue. Towards the applicability in robotic surgery scenarios, this study will be complemented with future experiments involving different indentation velocities to investigate the effects of viscoelasticity in the perception of stiffness. According to the proposed spike-based encoding paradigm, tactile displays installed on robotic manipulators are expected to allow surgeons to feel in real time the stiffness of human tissues, while force information is collected by force sensors installed on the robotic end effectors.

The tactile device herein presented is grounded on a novel neuromorphic discrimination mechanism for the recognition of the stiffness of rubber samples. Accordingly, a sequence of vibrotactile pulses are delivered on the skin surface, resembling the neural spikes produced by the mechanoreceptive endings underneath. Hence, the proposed strategy for encoding haptic data is non-homologous to physiological perception via the own fingers. The assumptions made in proposing such a strategy for the development of sensory substitution and augmentation devices were very strong. As a matter of fact, tactile devices are based on cognitive discrimination mechanisms [59] that are slow and require efforts when compared to natural perceptual processes, thus demanding a certain familiarization before allowing the perception of information in a reliable manner [10]. Physiological perception, achieved with the classical senses, allows the immediate discrimination of sensations, grounded on specialized neuronal architectures and on the training undertaken during the whole past life of a subject, and has important implications for the usability of a device in real-world applications. In this framework, we had evidence that the proposed neuromorphic model enabled the participants in our experiments to perceive the stiffness of materials in an intuitive manner. Further studies are however required to evaluate the degree of embodiment of the provided neuromorphic haptic perception, in comparison to the natural perceptual mechanisms of the somatosensory system. For applications that may encounter some limitations in delivering stimuli to the skin, we envisage the possibility to convert the trains of spikes in audio data. In this respect, a proof of feasibility is provided by electrophysiological experimental set-ups, that typically have loudspeakers or headsets to convert the recorded neural signals into audio data in real-time (with auditory patterns that may help the experimenter in identifying, as an example, a specific neuron type). Moreover, some ongoing initiatives, aiming at converting neural data into pleasant music and rhythms (see, e.g., [60]), provide additional evidence regarding the feasibility of achieving a meaningful auditory representation of spikes, as an alternative to haptic interfaces.

## Figures and Tables

**Table 1:** Normalized stiffness variations of the selected pairs of stimuli.

	Stimulus 1	Stimulus 2	Normalized Δstiffness $\frac{2\left(Stiffness2\;–\;Stiffness1\right)}{\left(Stiffness1+\;Stiffness2\right)}$
1	Econ 80	Ecoflex 30	−1.88
2	Pdms	Dragon Skin FX	−1.61
3	Pdms	Dragon Skin 10	−1.28
4	Smooth Sil 950	Dragon Skin 10	−1.04
5	Dragon Skin 10	Dragon Skin FX	−0.67
6	Pdms	Smooth Sil 950	−0.37
7	Smooth Sil 950	Pdms	0.37
8	Dragon Skin FX	Dragon Skin 10	0.67
9	Dragon Skin 10	Smooth Sil 950	1.04
10	Dragon Skin 10	Pdms	1.28
11	Dragon Skin FX	Pdms	1.61
12	Ecoflex 30	Econ 80	1.88

**Table 2:** Correspondence between the stiffness of the experimented stimuli and the inter-spike interval at the onset of the indentation as a result of the implemented neuromorphic haptic encoding strategy.

	Stiffness ± std (N/mm)	Mean ISI ± std (s)
Econ 80	78.9 ± 4.9	0.012 ± 0.001
PDMS	34.5 ± 0.2	0.0179 ± 0.002
Smooth Sil 950	23.8 ± 0.7	0.019 ± 0.002
Dragon Skin 10	7.5 ± 0.1	0.039 ± 0.004
Dragon Skin FX	3.7 ± 0.1	0.041 ± 0.004
EcoFlex 30	2.5 ± 0.1	0.043 ± 0.008

## References

[1] Kandel E.R., Schwartz J.H., Jessell T.M. (2000). Principles of Neural Science.

[2] Jones L.A., Lederman S.J., Jones L.A., Lederman S.J. (2006). Tactile sensing. Human Hand Function.

[3] Robles-De-La-Torre G. (2006). The importance of the sense of touch in virtual and real environments. IEEE Multimed..

[4] Bensmaia S.J., Miller L.E. (2014). Restoring sensorimotor function through intracortical interfaces: Progress and looming challenges. Nat. Rev. Neurosci..

[5] Bach-y-Rita P. (1970). Neurophysiological basis of a tactile vision-substitution system. IEEE Trans. Man-Mach. Syst..

[6] Bach-y-Rita P., Collins C.C., Saunders F.A., White B., Scadden L. (1969). Vision substitution by tactile image projection. Nature.

[7] Bach-y-Rita P., Tyler M.E., Kaczmarek K.A. (2003). Seeing with the brain. Int. J. Hum.-Comput. Interact..

[8] Bach-y-Rita P., Kaczmarek K.A., Tyler M.E., Garcia-Lara J. (1998). Form perception with a 49-point electrotactile stimulus array on the tongue: A technical note. J. Rehabilit. Res. Dev..

[9] Kaczmarek K.A., Bach-Y-Rita P. (1995). Tactile displays. Virtual Environ. Adv. Interface Des..

[10] Deroy O., Auvray M. (2012). Reading the world through the skin and ears: A new perspective on sensory substitution. Front. Psychol..

[11] Auvray M., Myin E. (2009). Perception with compensatory devices: From sensory substitution to sensorimotor extension. Cogn. Sci..

[12] Sorgini F., Caliò R., Carrozza M.C., Oddo C.M. (2017). Haptic-assistive technologies for audition and vision sensory disabilities. Disabil. Rehabilit. Assist. Technol..

[13] Caldwell D.G., Tsagarakis N., Giesler C. An Integrated Tactile/Shear Feedback Array for Stimulation of Finger Mechanoreceptor. Proceedings of the IEEE International Conference on Robotics and Automation.

[14] Loscos C., Tecchia F., Frisoli A., Carrozzino M., Widenfeld H.R., Swapp D., Bergamasco M. (2004). The Museum of Pure Form: Touching Real Statues in an Immersive Virtual Museum.

[15] Jörntell H., Bengtsson F., Geborek P., Spanne A., Terekhov A.V., Hayward V. (2014). Segregation of tactile input features in neurons of the cuneate nucleus. Neuron.

[16] Hayward V., Cruz-Hernandez M. Tactile display device using distributed lateral skin stretch. Proceedings of the Haptic Interfaces for Virtual Environment and Teleoperator Systems Symposium.

[17] Hayward V. (2008). A brief taxonomy of tactile illusions and demonstrations that can be done in a hardware store. Brain Res. Bull..

[18] Tegin J., Wikander J. (2005). Tactile sensing in intelligent robotic manipulation—A review. Ind. Robot.

[19] Verrillo R.T. (1985). Psychophysics of vibrotactile stimulation. J. Acoust. Soc. Am..

[20] Vallbo Å.B., Johansson R.S. (1984). Properties of cutaneous mechanoreceptors in the human hand related to touch sensation. Hum. Neurobiol..

[21] Brewster S., Brown L.M. Tactons: Structured tactile messages for non-visual information display. Proceedings of the Fifth Conference on Australasian User Interface.

[22] Gunther E., O’Modhrain S. (2003). Cutaneous grooves: Composing for the sense of touch. J. New Music Res..

[23] Caldwell D.G., Tsagarakis N., Wardle A. Mechano thermo and proprioceptor feedback for integrated haptic feedback. Proceedings of the International Conference on Robotics and Automation.

[24] Dahiya R.S., Metta G., Valle M., Sandini G. (2010). Tactile sensing—From humans to humanoids. IEEE Trans. Robot..

[25] Chouvardas V.G., Miliou A.N., Hatalis M.K. (2008). Tactile displays: Overview and recent advances. Displays.

[26] Liu Y., Yu Y., Yang J., Inai Y., Wu J. Ability to recognize and identify the location of vibration stimulation on the fingers. Proceedings of the IEEE International Conference on Mechatronics and Automation.

[27] Sziebig G., Solvang B., Kiss C., Korondi P. Vibro-tactile feedback for vr systems. Proceedings of the 2nd Conference on Human System Interactions.

[28] Alahakone A.U., Senanayake S., Arosha M. Vibrotactile feedback systems: Current trends in rehabilitation, sports and information display. Proceedings of the IEEE/ASME International Conference on Advanced Intelligent Mechatronics.

[29] Choi S., Kuchenbecker K.J. (2013). Vibrotactile display: Perception, technology, and applications. Proc. IEEE.

[30] Sibert J., Cooper J., Covington C., Stefanovski A., Thompson D., Lindeman R.W. Vibrotactile feedback for enhanced control of urban search and rescue robots. Proceedings of the IEEE International Workshop on Safety, Security and Rescue Robotics.

[31] Yamamoto T., Abolhassani N., Jung S., Okamura A.M., Judkins T.N. (2012). Augmented reality and haptic interfaces for robot-assisted surgery. Int. J. Med. Robot. Comput. Assist. Surg..

[32] Pacchierotti C., Prattichizzo D., Kuchenbecker K.J. (2016). Cutaneous feedback of fingertip deformation and vibration for palpation in robotic surgery. IEEE Trans. Biomed. Eng..

[33] Van der Meijden O., Schijven M. (2009). The value of haptic feedback in conventional and robot-assisted minimal invasive surgery and virtual reality training: A current review. Surg. Endosc..

[34] Tiwana M.I., Redmond S.J., Lovell N.H. (2012). A review of tactile sensing technologies with applications in biomedical engineering. Sens. Actuators A Phys..

[35] Tavakoli M., Patel R.V., Moallem M. A force reflective master-slave system for minimally invasive surgery. Proceedings of the 2003 IEEE/RSJ International Conference on Intelligent Robots and Systems (IROS 2003).

[36] Eltaib M., Hewit J. (2003). Tactile sensing technology for minimal access surgery—A review. Mechatronics.

[37] Peirs J., Clijnen J., Reynaerts D., Van Brussel H., Herijgers P., Corteville B., Boone S. (2004). A micro optical force sensor for force feedback during minimally invasive robotic surgery. Sens. Actuators A Phys..

[38] Hu T., Castellanos A.E., Tholey G., Desai J.P. Real-time haptic feedback in laparoscopic tools for use in gastro-intestinal surgery. Proceedings of the International Conference on Medical Image Computing and Computer-Assisted Intervention.

[39] Franks J., Culjat M., King C.-H., Franco M., Bisley J., Grundfest W., Dutson E. (2008). Pneumatic balloon actuators for tactile feedback in robotic surgery. Ind. Robot.

[40] Oddo C.M., Raspopovic S., Artoni F., Mazzoni A., Spigler G., Petrini F., Giambattistelli F., Vecchio F., Miraglia F., Zollo L. (2016). Intraneural stimulation elicits discrimination of textural features by artificial fingertip in intact and amputee humans. eLife.

[41] Oddo C.M., Mazzoni A., Spanne A., Enander J.M.D., Mogensen H., Bengtsson F., Camboni D., Micera S., Jörntell H. (2017). Artificial spatiotemporal touch inputs reveal complementary decoding in neocortical neurons. Sci. Rep..

[42] Cipriani C., Segil J.L., Clemente F., Weir R.F., Edin B. (2014). Humans can integrate feedback of discrete events in their sensorimotor control of a robotic hand. Exp. Brain Res..

[43] Rongala U.B., Mazzoni A., Oddo C.M. (2017). Neuromorphic artificial touch for categorization of naturalistic textures. IEEE Trans. Neural Netw. Learn. Syst..

[44] Bicchi A., Scilingo E.P., Ricciardi E., Pietrini P. (2008). Tactile flow explains haptic counterparts of common visual illusions. Brain Res. Bull..

[45] Terekhov A.V., Hayward V. (2015). The brain uses extrasomatic information to estimate limb displacement. Proc. Biol. Sci..

[46] Schumann F., O’Regan J.K. (2017). Sensory augmentation: Integration of an auditory compass signal into human perception of space. Sci. Rep..

[47] Sorgini F., Mazzoni A., Massari L., Caliò R., Galassi C., Kukreja S., Sinibaldi E., Carrozza M., Oddo C. (2017). Encapsulation of piezoelectric transducers for sensory augmentation and substitution with wearable haptic devices. Micromachines.

[48] Sorgini F., Ghosh R., Huebotter J.F., Caliò R., Galassi C., Oddo C.M., Kukreja S.L. Design and preliminary evaluation of haptic devices for upper limb stimulation and integration within a virtual reality cave. Proceedings of the 2016 6th IEEE International Conference on Biomedical Robotics and Biomechatronics (BioRob).

[49] Izhikevich E.M. (2003). Simple model of spiking neurons. IEEE Trans. Neural Netw..

[50] Ulrich R., Miller J. (2004). Threshold estimation in two-alternative forced-choice (2AFC) tasks: The spearman-kärber method. Atten. Percept. Psychophys..

[51] Hedges L.V., Olkin I. (1985). Chapter 5—Estimation of a single effect size: Parametric and nonparametric methods. Statistical Methods for Meta-Analysis.

[52] Antfolk C., D’Alonzo M., Controzzi M., Lundborg G., Rosen B., Sebelius F., Cipriani C. (2013). Artificial redirection of sensation from prosthetic fingers to the phantom hand map on transradial amputees: Vibrotactile versus mechanotactile sensory feedback. IEEE Trans. Neural Syst. Rehabilit. Eng..

[53] Antfolk C., D’Alonzo M., Rosén B., Lundborg G., Sebelius F., Cipriani C. (2013). Sensory feedback in upper limb prosthetics. Expert Rev. Med. Devices.

[54] Kerdegari H., Kim Y., Prescott T.J. (2016). Head-mounted sensory augmentation device: Designing a tactile language. IEEE Trans. Haptics.

[55] Bertram C., Evans M.H., Javaid M., Stafford T., Prescott T. Sensory augmentation with distal touch: The tactile helmet project. Proceedings of the Conference on Biomimetic and Biohybrid Systems.

[56] Cassinelli A., Reynolds C., Ishikawa M. Augmenting spatial awareness with haptic radar. Proceedings of the 10th IEEE International Symposium on Wearable Computers.

[57] Van Erp J.B., Van Veen H.A., Jansen C., Dobbins T. (2005). Waypoint navigation with a vibrotactile waist belt. ACM Trans. Appl. Percept..

[58] K önig S.U., Schumann F., Keyser J., Goeke C., Krause C., Wache S., Lytochkin A., Ebert M., Brunsch V., Wahn B. (2016). Learning new sensorimotor contingencies: Effects of long-term use of sensory augmentation on the brain and conscious perception. PLoS ONE.

[59] Cuturi L.F., Aggius-Vella E., Campus C., Parmiggiani A., Gori M. (2016). From science to technology: Orientation and mobility in blind children and adults. Neurosci. Biobehav. Rev..

[60] Destexhe A., Foubert L. The Spikiss Project. http://cns.iaf.cnrs-gif.fr/spikiss.html.

